# Multivalent Calixarene Complexation of a Designed Pentameric Lectin

**DOI:** 10.1021/acs.biomac.3c01280

**Published:** 2024-01-16

**Authors:** Ronan J. Flood, Linda Cerofolini, Marco Fragai, Peter B. Crowley

**Affiliations:** † SSPC, Science Foundation Ireland Research Centre for Pharmaceuticals, School of Biological and Chemical Sciences, University of Galway, University Road, Galway H91 TK33, Ireland; ‡ Magnetic Resonance Center (CERM), University of Florence, Via L. Sacconi 6, 50019 Sesto, Fiorentino, Italy; § Consorzio Interuniversitario Risonanze Magnetiche di Metalloproteine (CIRMMP), Via L. Sacconi 6, 50019 Sesto, Fiorentino, Italy; ∥ Department of Chemistry “Ugo Schiff”, University of Florence, Via della Lastruccia 3, 50019 Sesto, Fiorentino, Italy

## Abstract

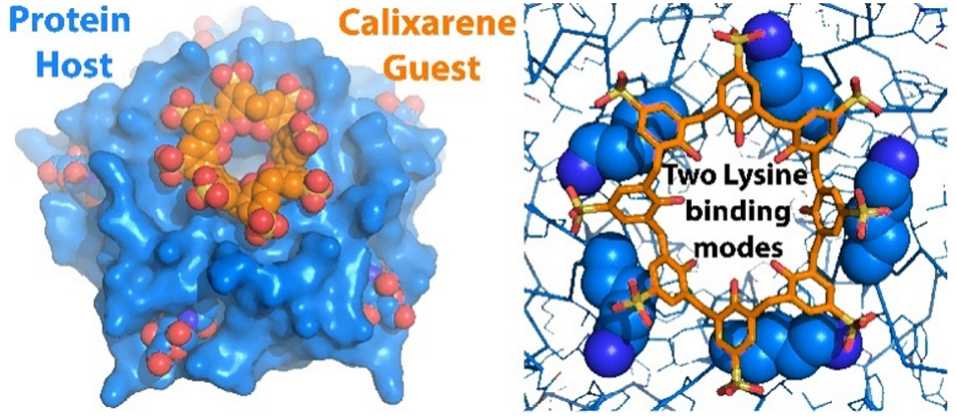

We describe complex formation between a designed pentameric β-propeller and the anionic macrocycle sulfonato-calix[8]arene (**sclx**_**8**_), as characterized by X-ray crystallography and NMR spectroscopy. Two crystal structures and ^15^N HSQC experiments reveal a single calixarene binding site in the concave pocket of the β-propeller toroid. Despite the symmetry mismatch between the pentameric protein and the octameric macrocycle, they form a high affinity multivalent complex, with the largest protein–calixarene interface observed to date. This system provides a platform for investigating multivalency.

## Introduction

Multivalency is central to biological interactions from bimolecular events such as protein–ligand complexation to multimolecular processes such as agglutination and cell–cell recognition.^1−4^ Here, we describe multivalent host–guest complexation between a designed pentameric lectin and the synthetic octameric macrocycle sulfonato-calix[8]arene (**sclx**_**8**_). The mechanism of multivalency contrasts with that employed in previous lectin-binding studies. Over 20 years ago, Bundle and co-workers reported an oligovalent carbohydrate ligand (*Starfish*) capable of dimerizing the pentameric binding subunit of Shiga-like toxin.^5^ A crystal structure (PDB 1QNU) revealed that *Starfish*, with five *arms* each bearing two trisaccharide *hands*, simultaneously engaged all five subunits of two toxin proteins. The X-ray data showed that the sugar-binding sites were each occupied by a single trisaccharide, while the ligand core and linkers were disordered. Since this seminal study, the impacts of the architecture and rigidity of multivalent carbohydrate ligands on lectin binding and/or agglutination have been variously tested.^6−10^ One approach to glyco-clustering for lectin complexation relies on calix[*n*]arene scaffolds.^11−15^ In the case of calix[4]arene, variants bearing one to four arms with carbohydrate hands have been used to complex lectins with up to five binding sites.^12,13^ A penta-glycosylated calix[5]arene was found to have 10^5^-fold tighter binding to Cholera toxin than the monomeric ganglioside GM1 oligosaccharide.^15^ These examples rely on the time-consuming and costly synthesis of multivalent carbohydrates. It transpires that the cost-effective calixarene scaffold can itself serve as a multivalent protein binder.

**Figure 1:**
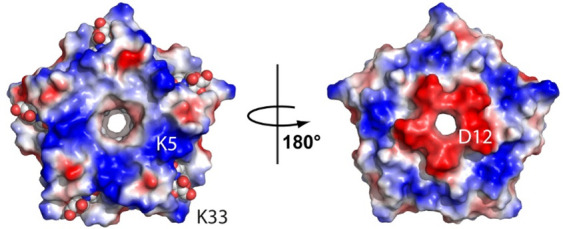
Electrostatic surface representation of Pent (based on PDB 8R3D and generated in PyMol) with cationic and anionic patches in blue and red, respectively. The funnel-like channel is evident. The wide end of the funnel includes Lys5, flanked by Asp21. The narrow end of the funnel protrudes through a convex surface comprising Pro11 and Asp12. GlcNAc is shown as spheres.

The β-propeller, with 4–12 blades, is a widespread toroidal fold with diverse functions ranging from ligand binding to catalysis and protein–protein interactions.^16−20^ Moreover, the repeat structure is amenable to multivalent interactions.^18,20^ Previously, we characterized the 6-bladed β-propeller *Ralstonia solanacearum* lectin (RSL) in complex with **sclx**_**8**_.^21−23^ The acidic RSL binds anionic **sclx**_**8**_ at pH 4, as evidenced by solution NMR spectroscopy. Co-crystallization of RSL and **sclx**_**8**_ is pH-dependent also, and at least four cocrystal forms are known.^21,22^ Here, we investigated **sclx**_**8**_ complexation with a designed 5-bladed β-propeller^18^ (PDB 5C2N), based on tachylectin-2. Each blade of this β-propeller is a 47-residue monomer of ∼5.2 kDa (yielding a pentamer of ∼26 kDa) that binds one equivalent of N-acetylglucosamine (GlcNAc). We were motivated to study this pentameric β-propeller, as it is unusual in the PDB, and owing to its lectin activity, it can be purified in a single step by affinity chromatography. The Asn33Lys mutation makes the protein cationic, with a calculated isoelectric point, p*I* ∼ 8 (Figure 1). For convenience, we named this protein *Pent*. Complex formation with **sclx**_**8**_ was characterized by X-ray crystallography and NMR spectroscopy. Both techniques reveal multivalent protein–calixarene binding,^24,25^ in which the protein is clearly the host and the macrocycle is the guest.^26^ Contrary to previous studies with the 6-bladed β-propeller,^21,22^ calix[8]arene binding is restricted to one site of Pent and the affinity is high (*K*_d_ ∼ μM).

## Experimental Section

### Sulfonato-calix[8]arene

Approximately 100 mM stock solutions of **sclx**_**8**_ (Tokyo Chemical Industry, S0471) were prepared in water and adjusted to pH 7.5.

### Protein Production and Purification

A pET-25(b+) vector containing the gene for 5c2n-N33K was produced by Genscript. Standard expression was performed in *Escherichia coli* BL21 (DE3) on an LB medium. Uniform ^15^N-labeled and ^13^C-, ^15^N-labeled protein samples were prepared using a two-step expression protocol.^27^ For selective labeling, the minimal medium contained 100 mg/L of ^15^N-Lys and 100 mg/L of the unlabeled amino acids (50 mg/L for Cys). Cell pellets were resuspended in 50 mM Tris-HCl, 150 mM NaCl, pH 7.5, with or without 50 mM MgCl_2_, and frozen overnight. The thawed cell suspension was further lysed by heating to 75 °C for 1 min, followed by incubation on ice for 10 min. Cell debris was removed by centrifugation and the protein was purified by affinity chromatography on GlcNAc-Agarose.^18^ The column was equilibrated with 50 mM Tris-HCl, 150 mM NaCl, with or without 50 mM MgCl_2_, pH 7.5, and elution was achieved with 50 mM Tris-HCl, 150 mM NaCl, 0.2 M GlcNAc pH 7.5. Pent-containing fractions were pooled and concentrated to ∼8 mM monomer in 20 mM Tris-HCl, 50 mM NaCl, pH 7.5, via ultrafiltration (Millipore, Amicon Ultra 3 kDa). Size exclusion chromatography (Figure S1) was performed by using an XK 16/70 column (1.6 cm diameter, 65 cm bed height) packed with Superdex 75 (GE Healthcare). Protein concentrations were determined by using ε_280_ = 13.9 mM^–1^ cm^–1^ for the monomer. Mass analysis was performed with an Agilent 6460 Triple Quadrupole LC/MS (Figure S2 and Table S1).

### Cocrystallization Trials

Mixtures of 1–2 mM Pent and 0–20 mM **sclx**_**8**_ were trialed with an Oryx8 Robot (Douglas Instruments) and a sparse matrix screen (JCSG++ HTS, Jena Bioscience) in 96-well MRC plates at 20 °C. GlcNAc (5–10 mM) was included in the trials to ensure complete occupancy of the sugar-binding sites in Pent. Hanging drop vapor diffusion trials in 24 well Greiner plates were performed also, testing 5–25% PEG of different average molecular weights, 0–200 mM MgCl_2_, 0–600 mM NaCl, and a variety of buffers from pH 4.6–8.8 (Table 1).

**Table 1:** Crystallization Conditions and Structure Properties

form	**sclx_8_** (mM)	% PEG/MWt	buffer (0.1 M)	salt (0.05 M)	space group	*a* × *b* × *c* (Å)	res (Å)	PDB id
I	2	10%/1k, 8k			*P*4_3_2_1_2	52 × 52 × 177	1.7	8R3B
II	1–2	15%/10k		MgCl_2_	*P*12_1_1	59 × 52 × 69	1.5	8R3C
	2	15%/10k	Bis-Tris pH 5.8		*P*1	97 × 107 × 112	1.9	
Pent only	2	15%/10k	Tris-HCl pH 8.8		*P*2_1_2_1_2_1_	53 × 59 × 72	1.7	8R3D

### X-ray Data Collection and Structure Determination

Crystals were transferred to reservoir solution supplemented with 25–30% glycerol and cryocooled in liquid nitrogen. Diffraction data were collected at 100 K at beamline PROXIMA-2A, SOLEIL synchrotron (France) with an Eiger X 9 M detector (Table S2). Data were processed using the autoPROC pipeline,^28^ with integration in XDS.^29^ The integrated intensities were scaled and merged in AIMLESS^30^ and POINTLESS.^31^ Structures were solved by molecular replacement in PHASER^32^ using one pentamer from PDB 5C2N(18) as the search model. The coordinates for **sclx**_**8**_ (ligand id EVB) and GlcNAc (ligand id NDG) were added in Coot.^33^ Iterative model building and refinement were performed in Coot and phenix.refine,^34^ respectively until no further improvements in the R_free_ or electron density were obtained. The structures, and associated structure factor amplitudes were deposited in the Protein Data Bank under the codes 8R3B, 8R3C, and 8R3D after validation in MolProbity.^35^ The statistics are listed in Table S2. Protein–**sclx**_**8**_ interface areas were measured in PDBe PISA.^36^

### NMR Characterization

A 2.5 mM uniformly ^13^C-, ^15^N-labeled Pent sample in 20 mM potassium phosphate, 50 mM NaCl, ∼10 mM GlcNAc, 10% D_2_O, pH 6.1 was used for resonance assignments. Samples for titration experiments comprised 0.25 mM Pent (either uniformly ^15^N-labeled or selectively ^15^N-lysine-labeled) in the same buffer and with μL aliquot additions of ∼10–100 mM **sclx**_**8**_. Solution NMR experiments for backbone resonance assignment [3D HNCA, HNCACB, CBCA(CO)NH, HNCO, HN(CA)CO]^37−39^ were recorded at 298 K on a Bruker AVANCE NEO NMR spectrometer, operating at 900 MHz ^1^H Larmor frequency (21.1 T), and equipped with a triple resonance 5 mm cryo-probe. 3D HNCA, HNCACB, CBCA(CO)NH spectra were acquired with nonuniform random sampling at 33%, 50% and 46%, respectively, and compressed-sensing reconstruction was used.^40^ The spectra were processed with Topspin 4.0.6, analyzed with CARA, and resonance assignment (Table S3) was aided by using the program ARTINA.^41^ For the NMR titrations, 2D ^1^H–^15^N HSQC watergate spectra were acquired at 30 °C with 8 scans and 64 increments on a Varian 600 MHz spectrometer equipped with a HCN cold probe.

## Results

### Pent Purification

Initial attempts to purify Pent via affinity chromatography failed. Improved purification of Pent was achieved with Mg^2+^-containing buffers.^42^ Sample purity was assessed by size exclusion chromatography (Figure S1). Samples prepared by affinity chromatography in standard buffer^18^ contained high molecular weight aggregates, as evidenced by elution at the column dead volume.^42^ In contrast, samples purified in the presence of MgCl_2_ resulted in a single peak in the size exclusion chromatogram. Affinity chromatography was optimal when the column was equilibrated in 50 mM Tris-HCl, 150 mM NaCl, and 50 mM MgCl_2_ at pH 7.5. The protein identity was confirmed by mass spectrometry, the measured mass of 5190.6 Da agreeing with the calculated mass of 5190.8 Da for the polypeptide lacking Met1 (Figure S2 and Table S1).

### Cocrystallization Trials

**Figure 2:**
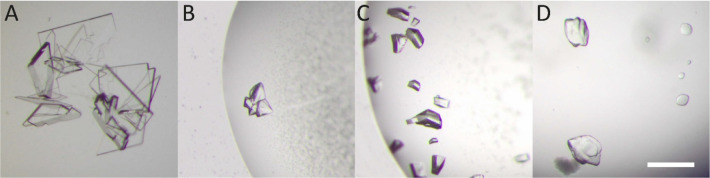
Cocrystals of Pent and **sclx**_**8**_ in space groups (A) *P*4_3_2_1_2, (B) *P*12_1_1, and (C) *P*1. (D) Crystals of Pent only. The scale bar is 100 μm.

A sparse matrix screen (JCSG++ HTS, Jena Bioscience) yielded one lead for Pent–**sclx**_**8**_ cocrystallization. Condition C12, containing 10% PEG 1000 and 10% PEG 8000, at 2 mM **sclx**_**8**_ yielded thin crystals of ∼100 μm in 3–5 days (Figure 2A). We tested the effect of the ionic strength on this condition by adding 0–200 mM MgCl_2_ or 0–600 mM NaCl. Buffers ranging from pH 4.6–8.8 and different PEG molecular weights were also tested. These efforts yielded three additional crystal types in 15% PEG 10000. Distorted ellipsoids were obtained with 50 mM MgCl_2_ (Figure 2B), while trapezoidal crystals grew at 100 mM Bis-Tris pH 5.8 (Figure 2C). An unusual, rounded morphology was obtained over 5–7 days at 100 mM Tris-HCl pH 8.8 (Figure 2D). Table 1 lists the crystallization conditions. All four crystal types were analyzed by X-ray diffraction at SOLEIL synchrotron (Table S2).

### Pent–**sclx_8_** Cocrystal Form I

**Figure 3:**
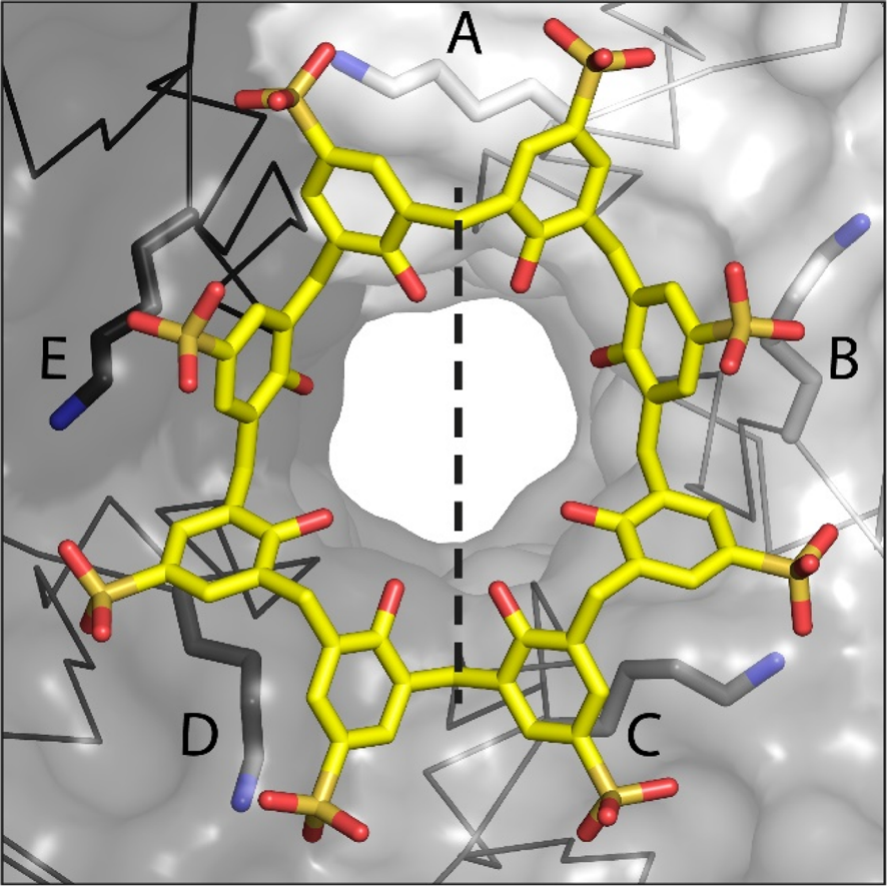
Detail of the Pent–**sclx**_**8**_ binding site. Each of the five protein subunits (A–E) binds the calixarene. The dashed line indicates the pseudo-*C*_2_ symmetry axis in the macrocycle. Two phenol-sulfonate units partially encapsulate Lys5 in chains A, C, and D. In contrast, Lys 5 is not encapsulated in chains B and E. Waters are omitted for clarity.

The thin plates (Figure 2A) yielded diffraction data extending to 1.7 Å resolution. The data were solved in the space group *P*4_3_2_1_2 with an asymmetric unit comprising one Pent and one **sclx**_**8**_. Contrary to the presumed existence of at least five binding sites, only one calixarene is bound to Pent. The presence of the calixarene is clear in the unbiased electron density map (obtained after molecular replacement and prior to including the calixarene coordinates in the model; Figure S3A). As is typical of β-propellers, Pent has a toroidal structure with a funnel-like central channel (Figure 1). The wide end of the funnel has the right dimensions to accommodate one calix[8]arene (Figure 3). In this binding mode, the phenol rim of the calixarene augments the water-filled channel of the toroid. Nevertheless, binding at this site is surprising considering the symmetry mismatch between the pentameric protein and the octameric calixarene. The calixarene adopts a pseudo-*C*_2_ symmetric conformation similar to the pleated loop^43^ but with two phenol-sulfonate units pointing out of the plane. In this conformation, the calixarene binds each of the five protein subunits, with interface areas ranging from 120 to 190 Å^2^ and a total interface area of 765 Å^2^ of the calixarene. This protein–calixarene interface is the largest observed to date, a consequence of multivalent complexation. Notwithstanding some side chain disorder (i.e., poor electron density for the C^ε^ and N^ζ^ atoms), each of the Lys5 residues is dominant, contributing on average ∼90 Å^2^ to the interface area. There are differences in binding, apparently due to the symmetry mismatch, such that three of the Lys5 residues are encapsulated, while two are not (Figure 3). The encapsulated lysines interact with the calixarene via both salt bridging a sulfonate and weak cation-π bonding to a phenol. The nonencapsulated side chains interact only via weak cation-π bonds. Binding at Lys5 is further interesting since it forms a salt bridge with the C-terminus Trp48, and it is flanked by Asp21 (Figure S4). These interactions dampen the cationic nature of the site.

**Figure 4:**
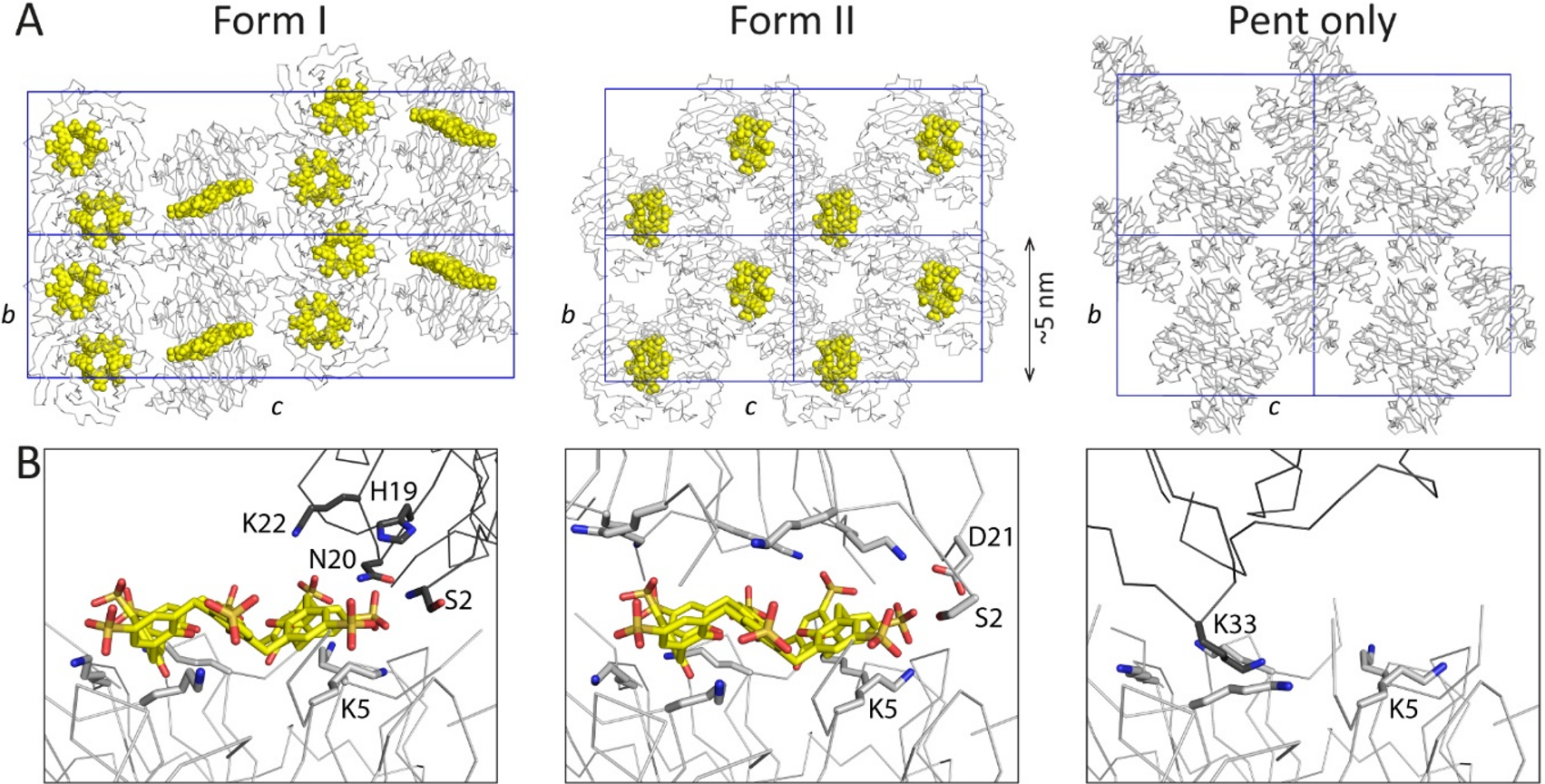
(A) Crystal packing in Pent–**sclx**_**8**_ and Pent only structures, with protein shown as the C^α^ trace in gray, **sclx**_**8**_ as yellow spheres, and unit cell axes in blue. (B) Details of the binding sites with interfacing side chains are shown as sticks. Symmetry mates are indicated as dark gray traces.

Considering crystal packing (Figure 4), there are two additional protein–calixarene interfaces. Two symmetry mates pack against the protein–calixarene assembly, forming interfaces (125 Å^2^) similar in size to the smallest interface in the multivalent site. Here, the dominant side chain is Asn20 (55 Å^2^). Cationic groups also contribute to calixarene complexation, including the N-terminus Ser2, Lys22, and to a minor extent His19.

### Pent–**sclx_8_** Cocrystal Form II

Despite their unusual morphology (Figure 2B), diffraction data extending to 1.6 Å resolution were collected from the crystals grown in the presence of MgCl_2_. This structure was solved in space group *P*12_1_1 with an asymmetric unit comprising two Pent molecules and one **sclx**_**8**_ (Figure 4). The calixarene, evident in the unbiased electron density map (Figure S3B), is sandwiched between two molecules of Pent arranged as a dimer. This dimer assembles via the concave pockets of each protein, with the Ser2 and Asp21 side chains on each subunit contributing to the Pent–Pent interface. Although the concave pocket is the calixarene binding site, due to steric constraints, only one **sclx**_**8**_ can be accommodated within the Pent dimer. One of the protein–calixarene interfaces is similar to form I, while the “capping” protein has fewer interactions with the calixarene and an ∼2-fold smaller interface area. The calixarene was refined at 60% occupancy and with high B-factors (∼50 Å^2^ vs ∼30 Å^2^ for the protein), which may be due to fluxionality of the macrocycle between the two binding sites available in the Pent–Pent dimer.

The crystals obtained in the presence of Bis-Tris at pH 5.8 (Figure 2C) diffracted to 2.0 Å resolution. This structure was solved in *P*1, but was not refined due to the complications arising from tNCS and an asymmetric unit comprising 16 × Pent (80 chains). Moreover, it is apparent from the data that this structure is equivalent to that of form II with a similar protein dimer hosting one calixarene (Figure S3C). The increased ionic strength of the crystallization conditions in form II (including 50 mM MgCl_2_ or 0.1 M Bis-Tris) versus form I may have altered the protein–calixarene assembly in favor of protein dimerization.

### Pent-Only Crystal Structure

The unusual crystals that grew in Tris-HCl at pH 8.8 (Figure 2D) diffracted to 1.7 Å resolution. These data were solved in space group *P*2_1_2_1_2_1_ with an asymmetric unit comprising one Pent. Despite the presence of 2 mM **sclx**_**8**_ during crystallization, there was no calixarene in this structure. This result can be interpreted in light of the crystallization pH, which is almost one unit above the calculated p*I* of Pent. Under these conditions, calixarene complexation is apparently switched off as the protein is anionic. This structure is further interesting as the crystal packing interfaces are distinct to those found in the Pent–**sclx**_**8**_ forms I and II. For example, the Pent dimer in form II does not occur in the Pent only structure or in PDB 5c2n. Furthermore, intramolecular noncovalent bonds such as the Lys5-Trp48 salt bridge are preserved in the Pent-only structure (Figure S4).

### NMR Analysis of Pent–**sclx_8_** Complexation

The Pent subunit has 47 residues (excluding Met 1), three of which are proline, at positions 11, 28, and 29. Resonance assignments were obtained from the analysis of triple-resonance spectra recorded on [U–^13^C, ^15^N] Pent in the presence of excess GlcNAc (Table S3). The program Artina^41^ aided the assignment process. All of the spin systems (except Pro28) were identified, and the backbone amide NH resonance was assigned for all residues. Some N-terminal resonances (Gly3 and Phe4) were split at 900 MHz, while the Lys5 and Asp21 signals were broad.

**Figure 5:**
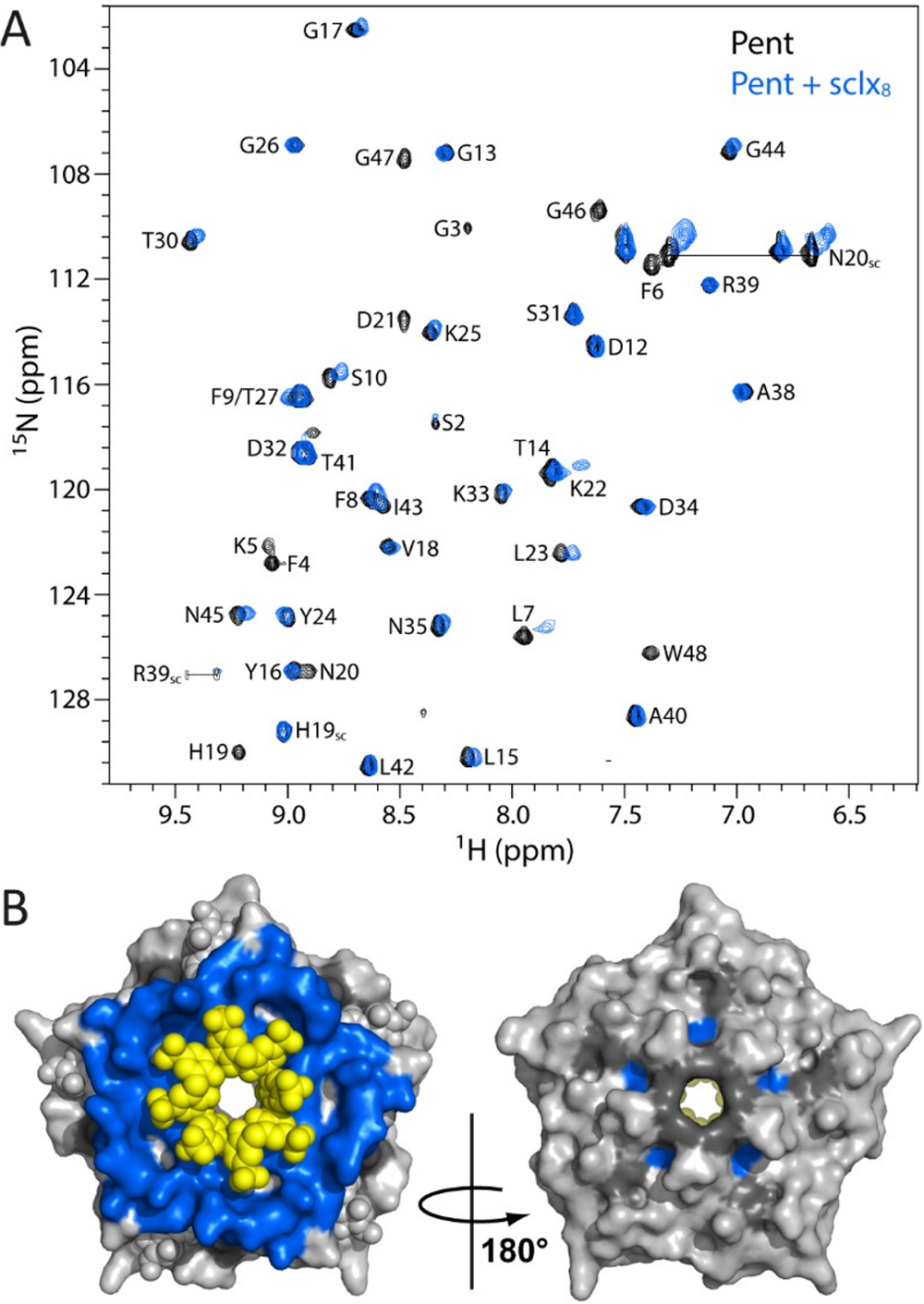
(A) The overlaid ^1^H–^15^N HSQC spectra of Pent in the absence (black contours) or presence of ∼2 equiv **sclx**_**8**_ (blue contours). (B) The Pent–**sclx**_**8**_ form I cocrystal structure with the protein and calixarene in surface and sphere representation, respectively. Blue corresponds to residues with significant effects in the HSQC, while gray is unaffected and dark gray is proline or unassigned.

Figure 5 shows the overlaid 2D ^1^H–^15^N HSQC spectra of Pent with and without the calixarene. At ∼2 equiv of **sclx**_**8**_, significant chemical shift perturbations and/or severe line broadening is evident for about half of the backbone amide resonances. The strongly affected amides are the N-terminal residues 2–10, the midsegment 19–23, and the C-terminal residues 46–48. The resonances with pronounced broadening are Phe4, Lys5, His19, Asn20, Asp21, Gly46, Gly47, and Trp48. The affected residues are mainly clustered around the Lys5/Trp48 pair and are fully consistent with the crystal structure of the Pent–**sclx**_**8**_ complex. A ^15^N-Lys-labeled Pent sample was also tested. Titration of the sample with **sclx**_**8**_ yielded clear-cut evidence of a slow-exchange process on the NMR time scale (Figure S5). These data suggest that the dissociation constant is *K*_d_ < 3 μM.^44^ Such tight binding is consistent with the large protein–calixarene interface observed in the crystal structures. Interestingly, the chemical shift perturbations of Lys22 are in the slow to intermediate exchange (Figure S5). This residue is peripheral to the main binding site and is involved in a crystal packing interaction with **sclx**_**8**_ in form I (Figure 4B). It is plausible that the calixarene binds transiently at this residue in solution. In contrast, the most solvent exposed lysine, Lys33, is minimally perturbed by **sclx**_**8**_. Compared to multivalent complexation at Lys5, the highly accessible but individual Lys33 is insufficient for calixarene binding.

## Discussion

The 6-bladed β-propeller RSL binds **sclx**_**8**_ with low affinity, cocrystallizing in at least four forms.^21,22^ These structures involve six different protein–calixarene interfaces, and the macrocycle is engaged to varying degrees as a molecular glue. In contrast, the 5-bladed β-propeller Pent binds **sclx**_**8**_ with high affinity at one well-defined site. What are the reasons for these different binding modes? The high affinity for Pent is explained on the basis of multivalent complexation with essentially five interfaces combined in one, yielding an interface area of ∼765 Å^2^. The largest interface in RSL–**sclx**_**8**_ utilizes ∼550 Å^2^ of the calixarene (PDB 6z60). In addition to multivalency, Coulombic interactions are favorable between cationic Pent and anionic **sclx**_**8**_ up to pH 7, while pH 4 or lower is required in the case of RSL. Another consideration is the subunit size. While RSL (∼29 kDa) and Pent (∼26 kDa) are similar in total mass, their subunits are markedly different. The RSL subunit (∼10 kDa) with an exposed surface area of ∼3900 Å^2^ is about twice the size of the Pent subunit (∼5 kDa, ∼2100 Å^2^). Consequently, Pent has fewer surface patches than does RSL for accommodating **sclx**_**8**_. Rather, the concave pocket arising from the β-propeller toroidal structure is the right size to tightly bind **sclx**_**8**_ (Figures 3 and 4). Apart from two small crystal packing junctions in form I, **sclx**_**8**_ does not function as a molecular glue in this system. This lack of glue activity is consistent with the calixarene being mostly concealed within the Pent pocket. Similarly, the NMR data suggest simple complex formation, rather than macrocycle-mediated oligomerization as occurs for monomeric cytochrome *c* (∼13 kDa).^45^

While **sclx**_**8**_ is a well-established host macrocycle in supramolecular chemistry,^26,46,47^ it behaves as a guest sitting in the host Pent. This hosting action of the protein is emphasized in crystal form II, where a Pent dimer encapsulates one **sclx**_**8**_ (Figures S3B and 4). The Pent–**sclx**_**8**_–Pent dimer is reminiscent of the assembly between a designed six-bladed symmetric β-propeller and a polyoxometalate (POM). In PDB 7ov7, a Cu-substituted Keggin-type POM is sandwiched between two β-propeller proteins that bind the copper ions via histidine side chains.^20^ The Pent–**sclx**_**8**_–Pent dimer is also reminiscent of the carbohydrate ligand *Starfish* complexed with two molecules of the pentameric binding subunit of Shiga-like toxin.^5^ In this classic example, the carbohydrate ligands were well-defined in the crystal structure, while the ligand core and linkers were disordered. The present study reveals that the *simple* calixarene scaffold can tightly bind a lectin (Figure 5). The sulfonates are an ∼15 Å distance from the GlcNAc, suggesting that a glyco-calix[8]arene with pentaethylene glycol linkers may be the right size for complexation. It remains to be seen whether such glyco-calix[8]arene conjugates are suitable multivalent ligands.

## Conclusion

The versatility of octameric **sclx**_**8**_ as a protein binder is further demonstrated through multivalent complexation of a pentameric β-propeller. Despite the symmetry mismatch, Pent and **sclx**_**8**_ form a high affinity (∼μM) complex, as evidenced by both crystallographic and NMR analyses. These results suggest an alternative pathway in the design of multivalent interfaces. Whereas previous strategies for lectin binding relied on synthetically challenging scaffolds with variable numbers of ligands, **sclx**_**8**_ is a cost-effective and promiscuous protein binder with multivalent capability. In this case, the multivalency arises from the aromatic core of the macrocycle adapting to lysine complexation via cation−π bonds, while the anionic rim forms salt bridges with the lysine ammonium groups. Further investigation of the **sclx**_**8**_–Pent complexation is underway to reveal other assembly modes.

## Supporting Information Available

Size exclusion chromatography and mass analysis of Pent. X-ray crystallography statistics and electron density maps. NMR resonance assignments for Pent and HSQC spectra of ^15^N-Lys-labeled protein plus **sclx_8_** (PDF)
