# Supplementary material for: Multivalent Calixarene Complexation of a Designed Pentameric Lectin
Source: Biomacromolecules. 2024 Jan 16;25(2):1303–9. doi: 10.1021/acs.biomac.3c01280 (PMC10865345; doi:10.1021/acs.biomac.3c01280)
Supplement: Supplementary file 1 — bm3c01280_si_001.pdf [file bm3c01280_si_001.pdf]

Supporting Information

Multivalent Calixarene Complexation of a Designed Pentameric Lectin

Ronan J. Flood,[a] Linda Cerofolini,[b,c,d] Marco Fragai,[b,c,d] Peter B. Crowley*,[a]

[a]SSPC, Science Foundation Ireland Research Centre for Pharmaceuticals, School of Biological and Chemical Sciences, University of Galway, University Road, Galway, H91 TK33, Ireland.

[b]Magnetic Resonance Center (CERM), University of Florence, Via L. Sacconi 6, 50019 Sesto Fiorentino, Italy.

[c]Consorzio Interuniversitario Risonanze Magnetiche di Metalloproteine (CIRMMP), Via L. Sacconi 6, 50019 Sesto Fiorentino, Italy.

[d]Department of Chemistry "Ugo Schiff", University of Florence, Via della Lastruccia 3, 50019 Sesto Fiorentino, Italy.

*correspondence to: peter.crowley@nuigalway.ie, +353 91 49 24 80

Contents

Item		Page
**Figure S1**	Size exclusion chromatography of Pent	2
**Figure S2**	ESI^+ mass spectrum of Pent	3
**Table S1**	Predicted and measured masses of Pent	3
**Table S2**	X-ray data collection, processing and refinement statistics	4
**Figure S3**	Unbiased electron density maps reveal location of $\mathbf{sclx_8}$ in Pent co-crystals	5
**Figure S4**	Salt bridge interaction between Lys5 and the carboxy terminus of Trp48	6
**Table S3**	Backbone resonance assignments for Pent	7
**Figure S5**	^1H–^{15}N HSQC spectra of ^{15}N-Lys-labelled Pent during $\mathbf{sclx_8}$ titration	8
**References**		8

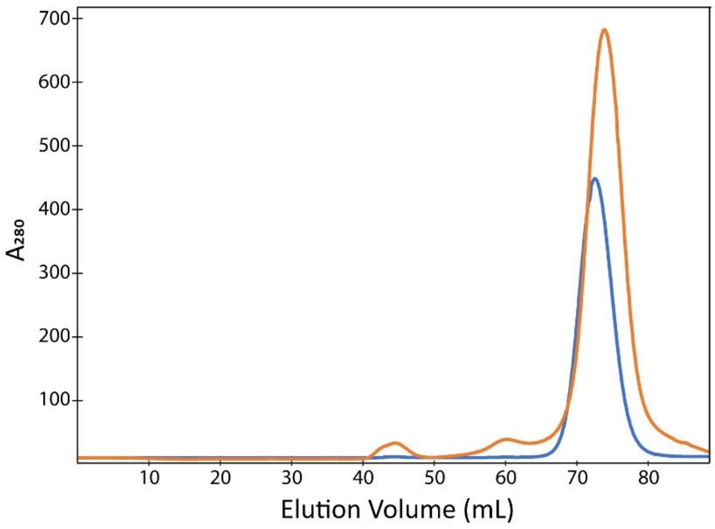

Figure S1. Size exclusion chromatography of Pent samples from two different preparations. The SEC chromatogram in blue, high purity, was obtained from a sample purified by affinity chromatography in buffer containing $MgCl_2$. The SEC chromatogram in orange, low purity, is the result after affinity chromatography without $MgCl_2$.

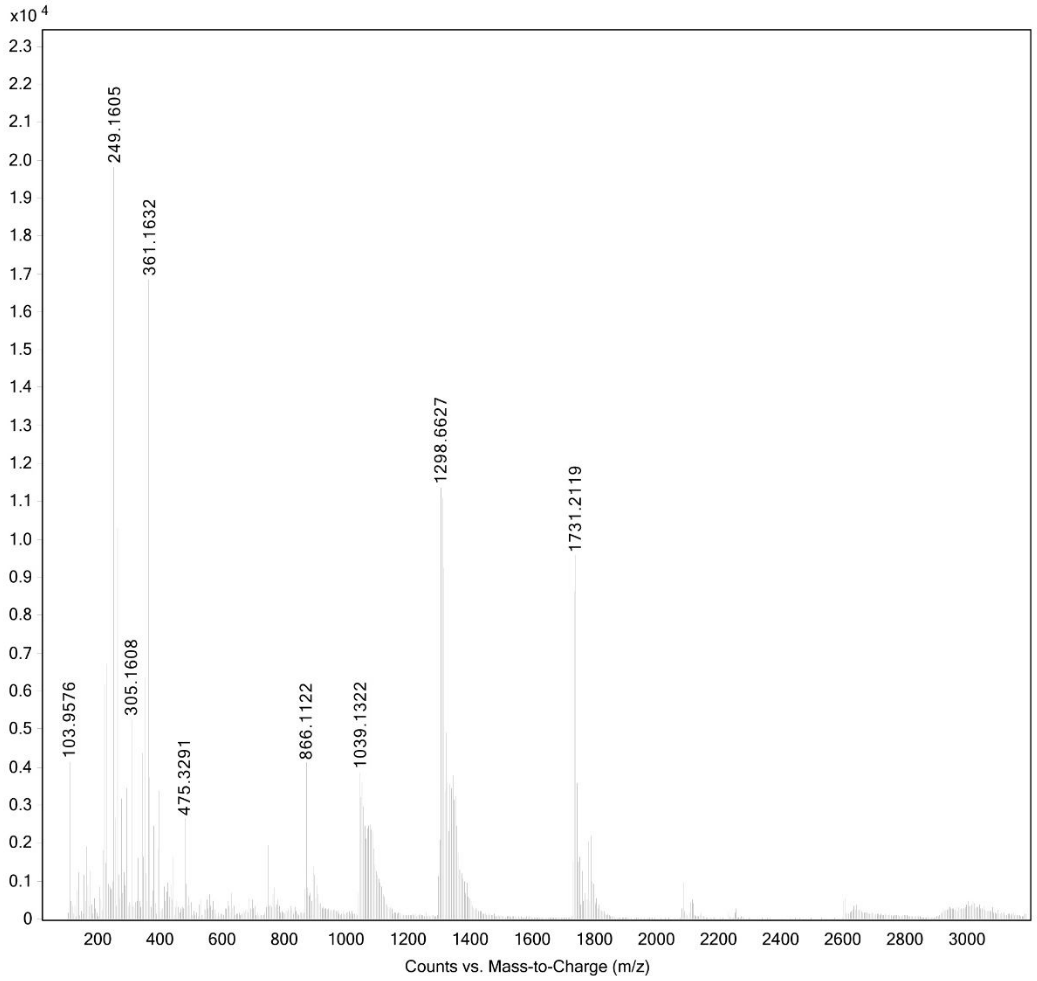

Figure S2. ESI^{+} mass spectrum of Pent.

Table S1. Predicted and measured masses from ESI^{+} mass spectrum using ESI-Prot.

m/z	charge	Molecular Weight (Da)	Error (Da)
866.1122	6+	5190.6289	0.007
1039.1322	5+	5190.6240	0.002
1298.6627	4+	5190.6212	-0.001
1731.2119	3+	5190.6135	-0.008
**Predicted MW (Da)**		5190.81	
**Deconvoluted MW (Da)**		5190.62	
**Standard deviation (Da)**		0.01	

Table S2. X-ray data collection, processing and refinement statistics for Pent – **sclx$_8$** co-crystals and Pent only crystals.

**Structure**	**Form I**	**Form II**	***P*1**	**Pent only**
Crystallization Conditions				
Protein (mM)	2	2	2	2
**sclx$_8$** (mM)	2	1	2	2
Precipitant	10% PEG 1,000<br>10% PEG 8,000	15% PEG 10,000	15% PEG 10,000	15% PEG 10,000
Buffer (0.1 M)	-	-	Bis-Tris pH 5.8	Tris-HCl pH 8.8
Salt	-	50 mM $MgCl_2$	-	-
Data Collection				
Light source	SOLEIL, PROXIMA-2A			
Wavelength (Å)	0.98013			
Space group	$P4_32_12$	$P12_11$	*P*1	$P2_12_12_1$
a, *b*, *c* (Å)	52.084<br>52.084<br>177.488	58.975<br>52.055<br>69.284	97.258<br>106.752<br>112.327	52.740<br>59.131<br>72.464
α, β, γ (°)	90.00<br>90.00<br>90.00	90.00<br>104.37<br>90.00	61.55<br>89.92<br>81.35	90.00<br>90.00<br>90.00
Resolution (Å)	49.98-1.66<br>(1.69-1.66)	57.13-1.58<br>(1.60-1.58)	95.84-1.99<br>(2.02-1.99)	45.81-1.71<br>(1.74-1.71)
# reflections	721110 (36791)	371965 (19371)	944429 (48805)	153453 (5526)
# unique reflections	30163 (1490)	55808 (2779)	263004 (12986)	25008 (1239)
Multiplicity	23.9 (24.7)	6.7 (7.0)	3.6 (3.8)	6.1 (4.5)
I/σ (I)	19.8 (2.3)	14.0 (2.2)	10.0 (2.1)	11.2 (2.2)
Completeness (%)	100.0 (100.0)	99.8 (100.0)	97.3 (96.4)	99.9 (100.0)
R_{meas} (%)	9.6 (160.4)	5.7 (66.4)	7.4 (64.1)	11.2 (69.6)
R_{pim} (%)	2.0 (32.1)	2.2 (25.0)	3.9 (32.9)	4.5 (32.3)
$CC_{1/2}$	99.9 (84.8)	99.8 (93.4)	99.8 (85.5)	99.7 (79.8)
Solvent content (%)	47	36	48	44
R_{work}	19.2	17.9		16.1
R_{free}	23.8	22.3		18.8
rmsd bonds (Å)	0.006	0.008		0.008
rmsd angles (°)	0.846	0.978		0.936
Pent	1	2		1
**sclx$_8$**	1	1		0
GlcNAc	5	10		5
Water	157	344		258
Avg. B-factor (Å^2)	29.47	33.67		19.91
clashscore	1.04	2.11		0.8
favoured regions	97.78	97.78		96.89
allowed regions	2.22	2.22		3.11
PDB code	8R3B	8R3C		8R3D

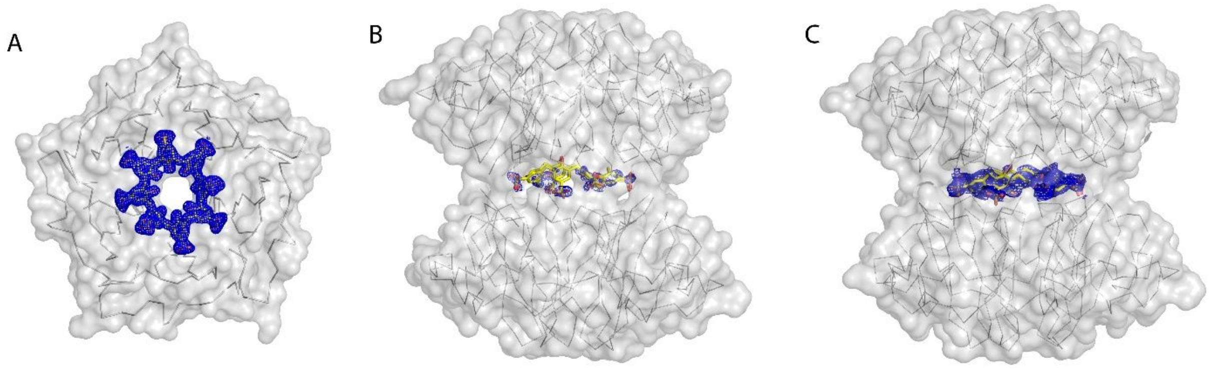

Figure S3. The unbiased 2Fo - Fc electron density maps, contoured at 1.0 σ (blue mesh), reveal **(A)** the unambiguous location of **sclx$_8$** in Form I, while **(B)** only the **sclx$_8$** sulfonates are clear in Form II, and **(C)** a calixarene-like blob is evident in the *P*1 dataset. The calixarene coordinates were added to the model after the map was prepared.

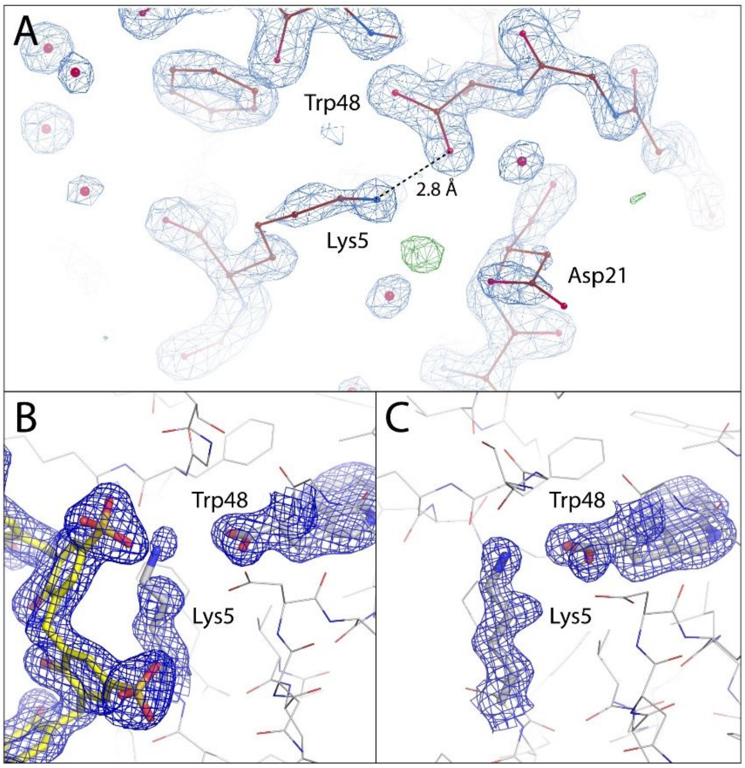

Figure S4. The salt bridge interaction between Lys5 and the carboxy terminus of Trp48, as evidenced in **(A)** PDB entry 5c2n (maps generated in PDB_REDO[1]), **(B)** Pent – **sclx$_8$** co-crystal form I, and **(C)** Pent only crystal structure. Panels (B,C) show the 2Fo - Fc electron density maps contoured at 1.0 σ.

Table S3. Resonance assignments for Pent in 20 mM potassium phosphate, 50 mM NaCl, 10 mM GlcNAc, 10% D_2O, pH 6.1 at 298 K and 900 MHz.*

Residue		$^1H^N$	N	C^α	C^β	C(O)
1	MET	8.50	128.62	54.00	33.35	178.50
2	SER	8.45	117.52	58.00	64.13	174.49
3	GLY	8.30	110.17	45.33		174.20
4	PHE	9.14	122.93	59.71	40.00	176.44
5	LYS	9.16	122.16	57.68	34.82	175.93
6	PHE	7.46	111.52	56.61	44.63	174.40
7	LEU	8.02	125.73	55.41	44.48	171.67
8	PHE	8.73	120.48	55.88	40.01	172.02
9	PHE	9.03	116.57	58.72	42.23	178.16
10	SER	8.90	115.82	55.15	62.73	175.13
11	PRO			64.48	31.40	176.44
12	ASP	7.72	114.63	53.53	40.17	177.08
13	GLY	8.39	107.22	45.20		174.23
14	THR	7.91	119.41	64.52	68.36	172.16
15	LEU	8.30	130.23	54.48	43.23	173.36
16	TYR	9.07	126.90	55.69	41.21	175.08
17	GLY	8.79	102.46	46.08		172.15
18	VAL	8.65	122.23	60.44	34.02	174.39
19	HIS	9.31	129.91	56.15	32.41	174.74
20	ASN	9.09	127.22	54.61	36.02	173.63
21	ASP	8.59	113.42	55.51		
22	LYS	7.91	119.53	55.67	35.54	173.33
23	LEU	7.85	122.44	53.14	43.11	173.75
24	TYR	9.08	124.98	56.66	44.24	174.90
25	LYS	8.46	114.11	53.72	35.30	174.99
26	GLY	9.06	106.95	45.32		173.63
27	THR	9.05	116.45	61.52	67.62	174.13
28	PRO					
29	PRO			62.55	32.50	177.77
30	THR	9.54	110.61	62.03	70.07	175.16
31	SER	7.81	113.35	57.50	65.32	172.76
32	ASP	9.03	118.38	55.50	39.24	176.54
33	LYS	8.17	120.57	56.08	31.99	175.36
34	ASP	7.54	120.64	52.96	41.53	174.86
35	ASN	8.46	125.56	52.36	36.73	174.56
36	TRP	7.54	130.60	63.33	30.43	177.69
37	LEU	9.08	118.05	57.17	41.68	178.06
38	ALA	7.04	116.26	53.44	18.59	178.18
39	ARG	7.22	112.36	54.48	30.55	175.89
40	ALA	7.58	128.75	52.25	17.41	176.26
41	THR	9.01	118.87	62.77	69.87	173.62
42	LEU	8.75	130.55	55.57	41.47	175.71

43	ILE	8.66	120.72	60.55	38.45	174.43
44	GLY	7.09	107.21	48.47		172.59
45	ASN	9.34	124.99	53.25	39.70	174.41
46	GLY	7.66	109.10	45.64		172.63
47	GLY	8.56	107.12	45.57		173.43
48	TRP	7.42	126.08	59.29	30.40	182.11

*The sample used for resonance assignment contained weak signals for Met1.

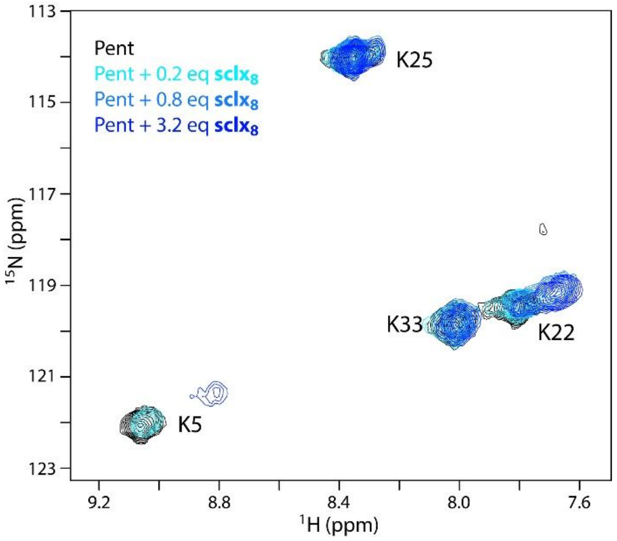

Figure S5. Overlaid ^{1}H–^{15}N HSQC spectra of ^{15}N-Lys-labelled Pent during **sclx$_8$** titration. Black contours correspond to the pure protein. The blue scale corresponds to the **sclx$_8$** concentration.

References

1. Joosten, R. P.; Long, F.; Murshudov, G. N.; Perrakis, A. The PDB_REDO server for macromolecular structure model optimization. *IUCrJ* **2014**, *1* (4), 213–220.
